# CT textural analysis of gastric cancer: correlations with immunohistochemical biomarkers

**DOI:** 10.1038/s41598-018-30352-6

**Published:** 2018-08-07

**Authors:** Shunli Liu, Hua Shi, Changfeng Ji, Wenxian Guan, Ling Chen, Yingshi Sun, Lei Tang, Yue Guan, Weifeng Li, Yun Ge, Jian He, Song Liu, Zhengyang Zhou

**Affiliations:** 1 Department of Radiology, Nanjing Drum Tower Hospital, The Affiliated Hospital of Nanjing University Medical School, Nanjing, 210008 China; 2 Department of Gastrointestinal Surgery, Nanjing Drum Tower Hospital, The Affiliated Hospital of Nanjing University Medical School, Nanjing, 210008 China; 3 Department of Pathology, Nanjing Drum Tower Hospital, The Affiliated Hospital of Nanjing University Medical School, Nanjing, 210008 China; 4 Department of Radiology, Peking University Cancer Hospital & Institute, Beijing, 100142 China; 5 School of Electronic Science and Engineering, Nanjing University, Nanjing, 210046 China

## Abstract

To investigate the ability of CT texture analysis to assess and predict the expression statuses of E-cadherin, Ki67, VEGFR2 and EGFR in gastric cancers, the enhanced CT images of 139 patients with gastric cancer were retrospectively reviewed. The region of interest was manually drawn along the margin of the lesion on the largest slice in the arterial and venous phases, which yielded a series of texture parameters. Our results showed that the standard deviation, width, entropy, entropy (H), correlation and contrast from the arterial and venous phases were significantly correlated with the E-cadherin expression level in gastric cancers (all P < 0.05). The skewness from the arterial phase and the mean and autocorrelation from the venous phase were negatively correlated with the Ki67 expression level in gastric cancers (all P < 0.05). The width, entropy and contrast from the venous phase were positively correlated with the VEGFR2 expression level in gastric cancers (all P < 0.05). No significant correlation was found between the texture features and EGFR expression level. CT texture analysis, which had areas under the receiver operating characteristic curve (AUCs) ranging from 0.612 to 0.715, holds promise in predicting E-cadherin, Ki67 and VEGFR2 expression levels in gastric cancers.

## Introduction

Gastric cancer is the fifth most common cancer and the third leading cause of cancer death worldwide, although the incidence and mortality rates have been recently declining^1^.

Most gastric cancer patients have an advanced stage of disease at the time of diagnosis, and the treatment options are limited, especially for patients with an M1 or a T4b stage^2^. Therefore, exploring new therapeutic options and identifying subgroups of patients who may benefit from special treatments has been a focal point of research. Great effort should be made in the research of biological molecular markers to determine the ability of tumour migration, proliferation and angiogenesis in gastric cancer.

E-cadherin is the main adhesion molecule of epithelia and has been implicated in carcinogenesis due to its frequent loss in human epithelial cancers^3^. According to previous studies, E-cadherin plays a vital role in the infiltration and metastasis of gastric cancer, and a negative expression of E-cadherin might be a predictive factor for a poor prognosis for gastric cancer^4^. Ki67 is a nuclear protein involved in cell proliferation regulation and is expressed in all phases of the cell cycle, except for the G0 phase^5^. Ki67 is usually recognized as a useful marker for the proliferation of tumour cells and has been a valuable prognostic and predictive marker for gastric cancer^6,7^. VEGFR2, the receptor of vascular endothelial growth factor (VEGF), is principally responsible for mediating the mitogenic-, angiogenic- and permeability-enhancing effects of VEGF and potentially plays a role in stimulating tumour growth and metastasis^8^. The expression of VEGFR2 might be a prognostic factor for gastric cancer, and the blockage of VEGFR2 in metastatic gastric cancers that progressed after fluoropyrimidine-based or platinum-based first-line chemotherapy has shown a survival benefit as a second-line treatment option^9,10^. It has been demonstrated that EGFR, a receptor tyrosine kinase, phosphorylates and regulates numerous cellular proteins and initiates several signal transduction cascades, leading to cell proliferation, migration, invasion, metastasis, angiogenesis and inhibition of apoptosis^11^. The EGFR expression level in gastric cancer is closely related to the incidence and development of gastric cancer, and it can provide a theoretical basis for the targeted therapy of gastric cancer positive for EGFR expression^12,13^.

Currently, immunohistochemistry in surgical specimens is the gold standard to assess the status of the above biomarkers, but it is not suitable for gastric cancer patients with distant metastasis who lose the opportunity to undergo surgical resection. Tumour specimens obtained from endoscopic biopsy are also available for immunohistochemistry, but this process involves an invasive procedure that includes unavoidable sample errors. Endoscopic ultrasonography (EUS) and multi-detector row computed tomography (MDCT) are the main modalities used for detecting and assessing gastric cancer preoperatively. In addition, magnetic resonance imaging (MRI) also has shown a potential value, owing to its high soft tissue resolution and multiple sequences imaging, but it still has not been widely applied in clinical practice for the diagnosis and evaluation of gastric cancer preoperatively. EUS plays an important role in the preoperative T staging of gastric cancer, especially in distinguishing mucosal and submucosal cancers^14^. However, EUS is an invasive examination and deeply relies on the operator’s experience. CT and MRI are both non-invasive modalities that can objectively assess the lesion and its adjacent structures. Compared with MRI, CT imaging is a less time-consuming examination and is less susceptible to respiratory artefacts^15^. Therefore, CT is the most widely used preoperative staging modality in gastric cancer at present^16^.

CT texture analysis is an adjunct tool involving the extraction of a large number of quantitative features from CT images to reflect the distribution and relationship of pixels. CT texture analysis not only detects subtle differences in pixels that cannot be recognized by the human eye but also assesses tumour heterogeneity, indirectly providing information of the tumour microenvironment^17^.

CT tumour texture analysis has shown promise in predicting the pathologic features, overall survival and response to therapy in various tumours composing gastric cancer^18–24^. Giganti *et al*. demonstrated that pre-treatment CT texture analysis might be a good prognostic biomarker and provide valuable information regarding the response rate to neo-adjuvant therapy, reflecting the aggressiveness and risk stratification for gastric cancer^21,23^. Our previous study also suggested that CT texture analyses held great potential in predicting differentiation degrees, the Lauren classification score and vascular invasion status of gastric cancers. However, the correlation between CT texture features and immunohistochemical biomarkers in gastric cancer has not been documented.

This study aimed to investigate the ability of CT texture analysis to assess and predict the expression levels of immunohistochemical biomarkers, including E-cadherin, Ki67, VEGFR2 and EGFR, in gastric cancer.

## Results

### The expression levels of immunohistochemical biomarkers

**Figure 1:**
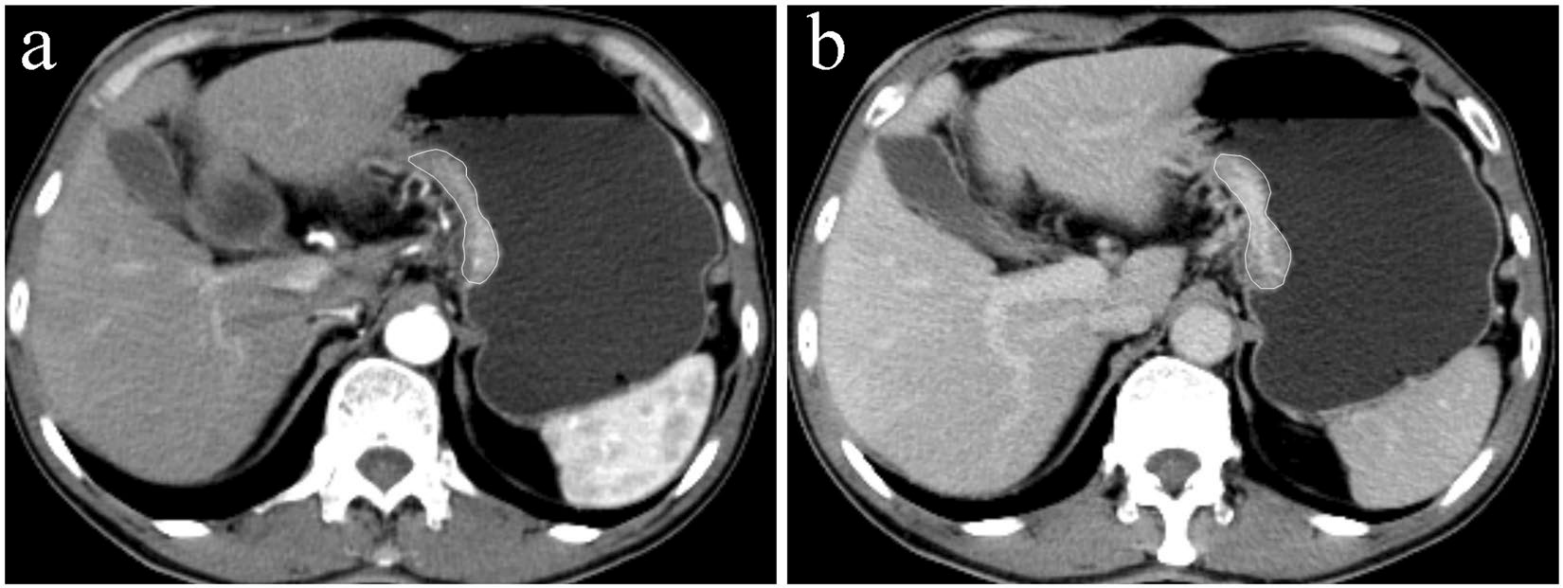
A 66-year-old man with poorly differentiated gastric adenocarcinoma positive for E-cadherin expression (+++), negative for Ki67 expression (expression index: 50%), weakly positive for VEGFR2 expression and weakly positive for EGFR expression. Axial CT images in the (a) arterial and (**b**) venous phases show a thickened wall with remarkable enhancement in the lesser curvature of the stomach. Note the region of interest (ROI) covering the largest slice of the lesion.

**Figure 2:**
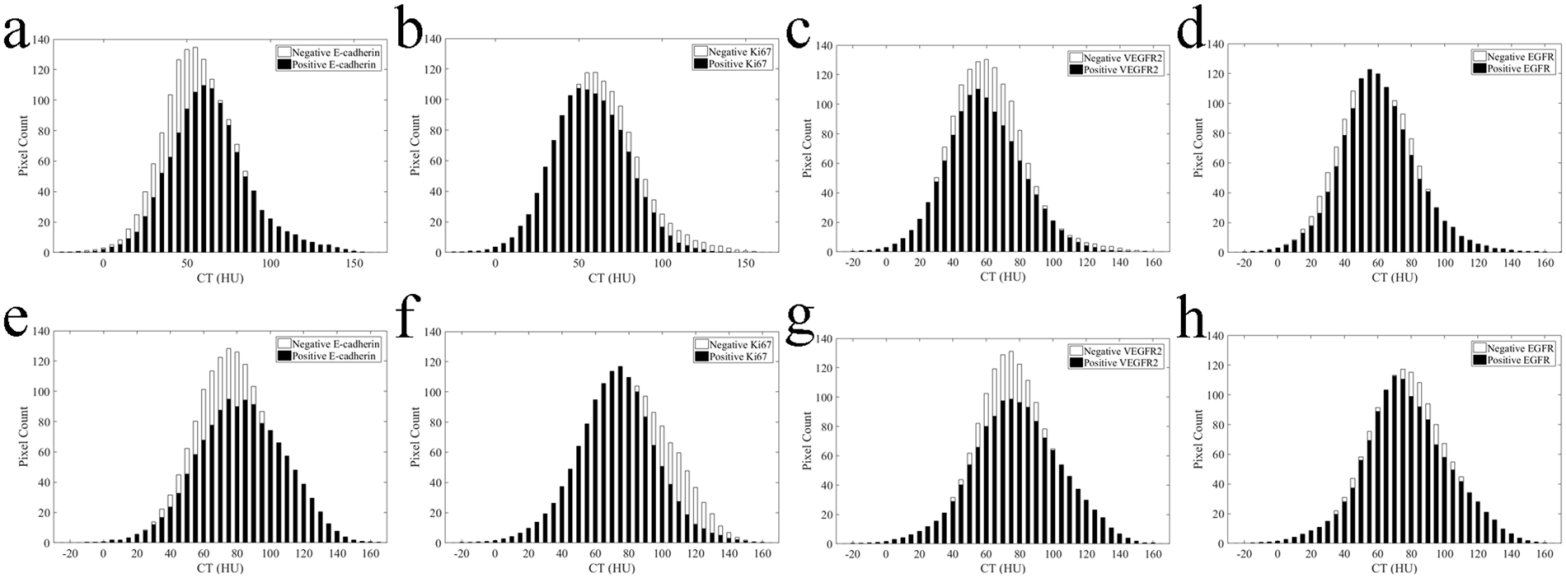
Averaged CT histograms derived from the arterial (**a,b,c,d**) and venous (**e,f,g,h**) phases show different distributions of pixel intensity in gastric cancers with different E-cadherin, Ki67, VEGFR2 and EGFR expression levels.

In our cohort, the rates of gastric cancers positive for E-cadherin, Ki67, VEGFR2 and EGFR expressions were 34.3% (35/102), 51.5% (70/136), 47.7% (63/132) and 32.8% (44/134), respectively. The ROI (region of interest) drawing and the averaged CT histograms in gastric cancers with different E-cadherin, Ki67, VEGFR2 and EGFR expression levels in the arterial and venous phases are shown in Figs 1 and 2, respectively.

### CT texture analysis in assessing E-cadherin expression in gastric cancer

Univariate analysis showed that the standard deviation (SD), width, entropy, entropy (H), correlation and contrast derived from the arterial and venous phases differed significantly between gastric cancers with positive and negative E-cadherin expressions (all P < 0.05). Spearman’s correlation test showed significant correlations between the above parameters and E-cadherin expression in gastric cancers (r = −0.250–0.327, all P < 0.05) (Table 1). Receiver operating characteristic (ROC) analysis showed that the above parameters could distinguish gastric cancers with positive E-cadherin expression from those with negative E-cadherin expression, and the area under the receiver operating characteristic curve (AUC) for these parameters ranged from 0.613–0.715 (all P < 0.05) (Table 2).

**Table 1:** Correlations between CT texture parameters and immunohistochemical markers of gastric cancers.

Parameter	Phase	VEGFR2		E-cadherin		Ki67	
		r	P value	r	P value	r	P value
Mean	Arterial	−0.117	0.182	0.051	0.607	−0.038	0.664
SD	Arterial	0.015	0.867	0.313	0.001*	−0.091	0.291
Skewness	Arterial	0.021	0.808	−0.028	0.776	−0.188	0.028*
Kurtosis	Arterial	−0.110	0.208	−0.085	0.396	0.042	0.626
Width	Arterial	0.027	0.762	0.327	0.001*	−0.094	0.276
Entropy	Arterial	0.024	0.781	0.281	0.004*	−0.090	0.297
Entropy(H)	Arterial	−0.009	0.918	0.217	0.029*	−0.009	0.920
Correlation	Arterial	0.080	0.362	−0.250	0.011*	0.010	0.909
Autocorrelation	Arterial	−0.099	0.258	0.086	0.391	−0.009	0.915
Contrast	Arterial	0.010	0.906	0.306	0.002*	−0.083	0.337
Mean	Venous	−0.007	0.936	0.057	0.567	−0.212	0.013*
SD	Venous	0.184	0.035	0.261	0.008*	−0.144	0.094
Skewness	Venous	−0.084	0.337	−0.018	0.856	0.101	0.240
Kurtosis	Venous	−0.122	0.164	−0.102	0.307	−0.059	0.493
Width	Venous	0.195	0.025*	0.246	0.013*	−0.131	0.130
Entropy	Venous	0.213	0.014*	0.229	0.021*	−0.136	0.115
Entropy(H)	Venous	0.109	0.215	0.155	0.121	−0.070	0.420
Correlation	Venous	−0.087	0.323	−0.200	0.044*	0.044	0.611
Autocorrelation	Venous	0.027	0.761	0.078	0.433	−0.219	0.011*
Contrast	Venous	0.187	0.032*	0.259	0.008*	−0.148	0.086

Note: r: correlation coefficient; SD: standard deviation; *P < 0.05.

**Table 2:** The diagnostic performance of CT texture parameters in predicting E−cadherin expression level in gastric cancers.

Parameter	Phase	Cut-off	Sen (%)	Spe (%)	Acc (%)	AUC	P value
SD	Arterial	18.92	48.6	83.6	71.6	0.698	<0.001
Width^#^	Arterial	44.00	54.3	76.1	68.6	0.715	<0.001
Entropy	Arterial	4.28	48.6	85.1	72.6	0.687	<0.001
entropy (H)	Arterial	7.12	88.6	44.8	59.8	0.675	0.001
Correlation^a^	Arterial	44.84	88.6	41.8	57.8	0.685	<0.001
Autocorrelation	Arterial	166.98	91.4	35.8	54.9	0.613	0.047
Contrast	Arterial	19.07	51.4	80.6	70.6	0.702	<0.001
SD	Venous	19.32	62.9	65.7	64.7	0.662	0.005
Width^#^	Venous	54.00	45.7	77.6	66.7	0.651	0.009
Entropy	Venous	4.32	60.0	68.7	65.7	0.643	0.013
entropy(H)	Venous	7.53	82.9	44.8	57.9	0.635	0.018
Correlation^a^	Venous	28.20	65.7	64.2	64.7	0.651	0.008
Contrast	Venous	21.40	65.7	61.2	62.7	0.660	0.005

Note: Sen: sensitivity; Spe: specificity; Acc: accuracy; AUC: area under the receiver operating characteristic (ROC) curve; SD: standard deviation; ^#^HU (Hounsfield unit); ^a^ × 10^−3^.

### CT texture analysis in assessing Ki67 expression in gastric cancer

Univariate analysis showed that skewness derived from the arterial phase, mean and autocorrelation derived from the venous phase differed significantly between gastric cancers with positive Ki67 expression and those with negative Ki67 expression (all P < 0.05). Spearman’s correlation test showed that skewness derived from the arterial phase, mean and autocorrelation derived from the venous phase were negatively correlated with the Ki67 expression level in gastric cancer (r = −0.188 to −0.219, all P < 0.05) (Table 1). ROC analysis showed that skewness derived from the arterial phase, mean and autocorrelation derived from the venous phase could distinguish gastric cancers with positive Ki67 expression from those with negative Ki67 expression (AUC = 0.621–0.647, all P < 0.05) (Table 3).

**Table 3:** The diagnostic performance of CT texture parameters in predicting VEGFR2 and Ki67 expression levels in gastric cancers.

	Parameters	Cut-off	Sen (%)	Spe (%)	Acc (%)	AUC	P value
Ki67 + vs. −	Skewness (A)	−0.21	45.7	86.4	65.4	0.647	0.002
	Mean^#^ (V)	86.74	82.9	42.4	63.2	0.621	0.013
	Autocorrelation (V)	470.02	82.9	42.4	63.2	0.621	0.013
VEGFR2 + vs. −	SD (V)	20.81	41.3	81.2	62.1	0.612	0.022
	Entropy (V)	4.39	38.1	85.5	62.9	0.626	0.010
	Width^#^ (V)	53.00	42.9	79.7	62.1	0.620	0.014
	Contrast (V)	26.58	39.7	82.6	62.1	0.615	0.019

Note: A: arterial phase; V: venous phase; Sen: sensitivity; Spe: specificity; Acc: accuracy; AUC: area under the receiver operating characteristic (ROC) curve; +: positive expression; −: negative expression; SD: standard deviation; ^#^HU (Hounsfield unit).

### CT texture analysis in assessing VEGFR2 expression in gastric cancer

Univariate analysis showed that the SD, width, entropy and contrast derived from the venous phase differed significantly between gastric cancer with positive VEGFR2 expression and those with negative VEGFR2 expression (all P < 0.05). Spearman’s correlation test indicated that width, entropy and contrast derived from the venous phase were positively correlated with the VEGFR2 expression level in gastric cancers (r = 0.187–0.213, all P < 0.05) (Table 1). ROC analysis showed that SD, width, entropy and contrast derived from the venous phase could distinguish gastric cancers with positive VEGFR2 expression from those with negative VEGFR2 expression (AUC = 0.612–0.626, all P < 0.05) (Table 3).

### CT texture analysis in assessing EGFR expression in gastric cancer

None of the CT texture features differed significantly between gastric cancers with positive EGFR expression and those with negative EGFR expression.

### Inter-observer agreement in the measurement of CT texture parameters

As shown in Table 4, mean, entropy (H), correlation and autocorrelation showed excellent inter-observer agreement (ICC = 0.850–0.940), while SD, width, entropy and contrast showed good inter-observer agreement (ICC = 0.757–0.792). However, skewness and kurtosis showed moderate inter-observer agreement (ICC = 0.551–0.595).

**Table 4:** Inter-observer agreement of CT texture parameters of gastric cancers.

Parameter	ICC in arterial phase (95% CI)	ICC in venous phase (95% CI)
Mean	0.940 (0.907–0.961)	0.918 (0.874–0.947)
SD	0.778 (0.659–0.856)	0.757 (0.607–0.847)
Skewness	0.586 (0.363–0.731)	0.551 (0.310–0.708)
Kurtosis	0.578 (0.351–0.726)	0.595 (0.377–0.737)
Width	0.786 (0.671–0.861)	0.792 (0.680–0.865)
Entropy	0.772 (0.649–0.852)	0.744 (0.576–0.842)
Entropy(H)	0.850 (0.769–0.902)	0.878 (0.812–0.921)
Correlation	0.868 (0.797–0.914)	0.913 (0.867–0.944)
Autocorrelation	0.869 (0.799–0.915)	0.927 (0.888–0.953)
Contrast	0.790 (0.677–0.863)	0.780 (0.662–0.857)

Note: ICC: intra-class correlation coefficient; CI: confidence interval.

## Discussion

Our study verified the correlation between CT texture features and immunohistochemical biomarkers, including E-cadherin, Ki67, VEGFR2 and EGFR, in gastric cancers, which has not been previously reported by any study until now.

Our data showed that SD, width, entropy, entropy (H), correlation and contrast from the arterial and venous phases were significantly correlated with the expression of E-cadherin in gastric cancer. A lower SD and a shorter width indicated a more centralized distribution. Less entropy, less entropy (H), less contrast and a higher correlation implied a lower degree of chaos. Our data suggested that the grey-level distribution was more centralized and homogeneous in gastric cancers negative for E-cadherin expression. According to a previous study, CT texture analysis can be applied to assess histopathological features in gastric cancers. Gastric cancers of diffuse-type and with vascular invasion showed more homogeneous CT distributions (lower SD and entropy) than those of an intestinal-type and without vascular invasion^22^. Our data showed that SD and entropy were significantly lower in gastric cancers negative for E-cadherin expression than in those positive for E-cadherin expression. A previous meta-analysis^4^ also found that gastric cancers of diffuse-type and with vascular invasion had a significant decrease in the E-cadherin expression compared to those of intestinal-type and without vascular invasion (OR = 4.22 and 1.86, respectively), which provided indirect evidence to confirm our findings.

In our study, CT texture features performed well in distinguishing gastric cancers with positive expression of E-cadherin from those with negative expression of E-cadherin, with the AUCs ranging from 0.613 to 0.715. Compared with venous texture analysis, arterial texture analysis indicated a better predictive value. In addition, low expression of E-cadherin significantly predicted poor overall survival of gastric cancer patients (HR = 1.62, 95% CI: 1.34–1.96)^4^. Nevertheless, the prognostic value of CT texture analysis in gastric cancer still needs further investigation.

Our data showed skewness in the arterial phase and mean and autocorrelation in the venous phase were all negatively with correlated the expression of Ki67 in gastric cancer. More negative skewness indicated that the grey-level intensity of lesions was more partial to a relatively high-density range. A lower value of autocorrelation implied a lower extent of similarity of CT values, indicating a more heterogeneous distribution of grey-level intensity. According to previous studies^25^, CT arterial imaging might mainly reflect the blood supply and functional capillary density of gastric cancers, while venous imaging might reflect more dysfunctional neo-vessels and represent the distribution of contrast media in interstitial spaces. Our data suggested that the functional capillary density of lesions might be more abundant but dysfunctional neo-vessels might be reductive and more heterogeneous in gastric cancers positive for Ki67 expression. Few studies have reported the correlation of the CT performance and Ki67 expression in gastric carcinomas. Wang *et al*.^26^ found that the Ki67 expression of gastric carcinomas was significantly correlated with the thickness of the tumour and lymph node metastasis from the CT findings, indicating that traditional CT images provided restricted value in assessing the Ki67 expression level. Ki67 has been declared as a predictor of cell proliferation and malignant potential in human malignancies^27^. The Ki67 expression level has potential to be a prognostic biomarker in gastric cancer^6,7^. CT texture analysis might serve as a more sensitive tool to assess Ki67 expression in gastric cancer. In our study, skewness from the arterial phase and mean and autocorrelation from the venous phase performed well in predicting the expression level of Ki67 in gastric cancer, with AUCs ranging from 0.621 to 0.647.

We also found that there were higher values of SD, width, entropy and contrast in venous phase analysis in gastric cancer positive for VEGFR2 expression compared to in that negative for VEGFR2 expression, indicating that the distribution of dysfunctional neo-vessels might be more heterogeneous in lesions with positive VEGFR2 expression. VEGF and its receptor, VEGFR2, might compose the important receptor-ligand system in the process of angiogenesis in gastric cancer^9^. Additionally, targeting VEGFR2 was also considered a promising therapeutic strategy with regard to angiogenesis for gastric cancer^28^. SD, width, entropy and contrast derived from the venous phase proved useful in differentiating gastric cancers positive for VEGFR2 expression from those negative for VEGFR2 expression, despite of the weak predictive value with its highest AUC of 0.626.

Our data showed no significant association between the CT texture features and the EGFR expression in gastric cancers. There have been several studies on CT texture parameters related to the EGFR mutation status in lung adenocarcinomas^29,30^. Our preliminary findings suggested that CT texture analysis could have few potential applications in assessing the expression of EGFR in gastric cancer. Anyway, EGFR plays different roles in gastric cancers and lung adenocarcinomas^12,31^.

The inter-observer agreement of most texture parameters was well to excellent. The inter-observer agreement of skewness and kurtosis in both the arterial and venous phases was worse than that of the other parameters. Additionally, the inter-observer agreement of the second-order features seemed to be better than most first-order features, indicating that the second-order features are more reliable and repeatable.

## Limitations

Our study had a few limitations. First, the CT images of the gastric cancer patients were retrospectively obtained from several CT scanners. Nevertheless, a good inter-scanner agreement of the CT texture analysis was confirmed^32^. Second, unenhanced CT images were not enrolled into our study cohort for texture analysis because it is difficult to identify tumour margins exactly on unenhanced images, despite their potential value^33^. Finally, the largest slice of the lesion, rather than the whole lesion (i.e., contouring the lesion slice by slice), was selected for texture analysis, which might not have adequately represented the heterogeneous characteristics of the lesions. However, several previous studies suggested that the comparison of single-level and whole-tumour texture analyses of single lesions showed fairly comparable results^32,34^. In addition, the ROIs outlining the area of greatest enhancement could minimize the effect of necrotic tissues and reflect angiogenesis more intensively, but there might exist site-by-site biases when placing ROIs^35,36^. In contrast, largest-level texture analysis might accurately reflect the heterogeneity of the whole lesion and improve the repeatability and reproducibility of texture analysis.

## Conclusion

CT texture analysis might serve as a promising non-invasive diagnostic tool to predict immunohistochemical biomarkers, including E-cadherin, Ki67 and VEGFR2, in gastric cancers, indirectly reflecting the ability of tumour migration, proliferation and angiogenesis.

## Materials and Methods

### Patients

This retrospective study was approved by the ethics committee of the Institutional Review Board of Nanjing Drum Tower Hospital, and the requirement for informed consent was waived. We collected data from and analysed a total of 264 patients with a clinical diagnosis of gastric cancer between January 2014 and December 2016.

The inclusion criteria were: (1) with biopsy-proven gastric cancer; (2) with an identifiable lesion in contrast-enhanced CT images before surgery; (3) with a curative or palliative gastrectomy in our hospital.

The exclusion criteria were: (1) with any local or systematic treatment before surgery (n = 37); (2) with difficulty in outlining the margin due to it having too small of a size (the maximum diameter <1 cm) (n = 52); (3) with a previous partial gastrectomy (n = 19); (4) without a definite location or margin due to no contrast or poor contrast of the lesion in the enhanced CT image (n = 12); (5) without available immunohistochemical markers (n = 5).

Eventually, a total of 139 patients (age: 29–92 years; median age: 63 years) were enrolled in our study cohort, and the clinicopathological variables of the patients are presented in Table 5.

**Table 5:** Clinicopathological features of 139 patients with gastric cancer.

Feature	n (percentage)
Gender	
Male	103 (74.1%)
Female	36 (25.9%)
Age	
<60 years	41 (29.5%)
≥60 years	98 (70.5%)
Major location	
Cardia and fundus	51 (36.7%)
Body	34 (24.5%)
Antrum	54 (38.8%)
Siewert classification	
Siewert I	0 (0)
Siewert II	24 (47.1%)
Siewert III	27 (52.9%)
Main pathological type	
Tubular or papillary adenocarcinoma	111 (79.9%)
Poorly cohesive adenocarcinoma	22 (15.8%)
Signet-ring cell carcinoma	6 (4.3%)
Differentiation degree	
Poor	113 (81.3%)
Moderate/well	26 (18.7%)
Lauren classification	
Diffuse type	43 (30.9%)
Mixed type	39 (28.1%)
Intestinal type	57 (41.0%)
T stage	
≤T2	8 (5.7%)
T3	81 (58.3%)
T4	50 (36.0%)
N stage	
N0	20 (14.4%)
N1–3	119 (85.6%)
M stage	
M0	133 (95.7%)
M1	6 (4.3%)

Note: TNM stage was classified based on the 7th edition of the AJCC classification system.

### CT image acquisition

All CT examinations were performed on a 16- or 64-slice scanner (Light Speed Pro 16, VCT, or Discovery HD 750, GE Healthcare, US). Before the examination, all patients signed the informed consent, were requested to fast from solid food for at least six hours and received 600–1000 mL water orally to achieve gastric distension. All patients were in the supine position during the scan, and the scan covered the upper or the entire abdomen. The patients were trained to hold their breath during the CT scanning. Following the non-contrast scan, 1.5 mL/kg iodinated contrast agent (Omnipaque 350 mg I/mL, GE Healthcare, Shanghai, China) was injected intravenously, at a flow rate of 3.0 mL/s, by using a high-pressure syringe (Medrad Stellant CT injector system; One Medrad Drive Indianola, PA, US). Imaging was obtained with post-injection delays of 30 seconds and 70 seconds, corresponding to the arterial and venous phases, respectively, after initiation of the contrast material injection. The CT scanning parameters were: tube voltage: 120 kVp, tube current: 250–350 mA, slice thickness: 5 mm, slice interval: 5 mm, field of view: 35–50 cm, matrix: 512 × 512, rotation time: 0.7 s, and pitch: 1.375.

The mean interval between CT examination and surgery was 5 days (range: 1–10 days).

### CT texture analysis

Texture analysis was performed via an in-house software (Image Analyser 2.0, China). Manual recognition of gastric cancers was performed by a radiologist (S.L., with 5 years of experience in gastroenterology imaging) and confirmed by one abdominal radiologist (Z.Y.Z., with 11 years of experience in gastroenterology imaging), who were both blinded to the clinicopathological information of the patient. The lesions of gastric cancers on enhanced CT images were defined as focal thickening with obvious enhancement of the gastric wall. According to the literature^37^, focal thickening of the gastric wall by 6 mm or greater, compared with that of the adjacent gastric wall, was determined to be abnormal thickening and cancerous. A polygonal ROI (arterial phase CT images: mean area: 712.0 mm^2^, range: 94.1–2506.4 mm^2^; venous phase CT images: mean area: 716.6 mm^2^, range: 124.5–2459.2 mm^2^) was manually drawn along the margin of the lesion on the largest slice, carefully avoiding the gastric lumen and artefacts. The texture features were generated automatically from the above ROIs of CT images using the in-house software: (1) the first-order features describing the distribution of pixel intensity within the ROIs, including mean (mean pixel intensity), SD (standard deviation, spread of the distribution), skewness (asymmetry of a histogram), kurtosis (peakness or pointedness of a histogram), width (width between the 10th and 90th percentiles of intensity of a histogram) and entropy (irregularity or complexity of pixel intensities); (2) the second-order features were from the grey-level co-occurrence matrix (GLCM), including entropy (H), correlation, autocorrelation and contrast. The normalized GLCM element can describe the probability of a pair of grey levels that are separated by a certain distance in a certain direction, providing spatial information of the pixel distribution. In this study, the distance of the pair was one pixel, and the directions were 0°, 45°, 90° and 135°, respectively. We took the average values of GLCMs in the four directions as the final values of the second-order features. The formulas of the first-order entropy and the second-order features are shown in Supplementary Methods. Additionally, another abdominal radiologist (J.H., with 8 years of experience in gastroenterology imaging) performed manual recognition independently to evaluate the inter-observer variability of manual recognition in calculating the texture features of gastric cancers. All image analyses and calculations were performed separately for the CT images of the arterial and venous phases.

### Immunohistochemical evaluation

Immunohistochemical analysis was used to evaluate the expression of different markers, including E-cadherin, Ki67, VEGFR2 and EGFR. A pathologist (L.C.) with 7 years of experience in gastrointestinal pathology, who was blinded to the clinical information, including name, gender and age, of the patient, carefully reviewed the slides from each sample and assessed the extent of immunohistochemical staining.

The expression levels of E-cadherin were divided into four groups according to the percentages of E-cadherin-positive cells, as described previously^38^: 0: <10% of positive cells; 1+: 10–30% of positive cells; 2+: 30–60% of positive cells; 3+: >60% of positive cells. Samples with grade 0 or 1 + E-cadherin expression level were categorized as having negative staining^39^.

The Ki67 labelling index was estimated by evaluating the nuclear immunoreactivity of 1000 tumour cells in ten random fields at high magnification and calculating the percentage of cells with positive nuclear staining relative to all tumour cell nuclei in the area examined. More than 50% positive staining in the nucleus was defined as positive Ki67 staining^39^.

For the VEGFR2 expression level, the cases were scored based on the staining intensity and percentage of cells stained, similarly to previous studies^40^. Staining intensity was graded from 0 to 3 (0 = none; 1 = weak; 2 = moderate; and 3 = strong). The percentage of immunopositive cells was given a score from 0–3 (0: 0% immunopositive cells; 1: 1–25% immunopositive cells; 2: 26–50% immunopositive cells; 3: >50% immunopositive cells). According to the sum of the intensity and percentage, the expression of VEGFR2 was divided into three levels: scores of 0 and 2 were regarded as negative for VEGFR2 expression, scores of 3 and 4 as weakly positive, and scores of 5 and 6 as strongly positive.

The expression level of EGFR was graded from 0 to 3+: 0: no staining or membranous reactivity in <10% of tumour cells; 1+: weak, barely perceptible membranous reactivity in >10% of tumour cells; 2+: complete or basolateral membranous reactivity of either a non-uniform or weak intensity in at least 10% of cells; 3+: complete or basolateral membranous reactivity of a strong intensity in ≥10% of cells^13^. Patients with grade 0 were categorized as having negative staining.

### Statistical analyses

The normality distribution of CT texture parameters was evaluated by the Kolmogorov-Smirnov normality test. Based on the normality test results, univariate analysis was performed by the Mann-Whitney U test for CT texture parameters of gastric cancers with different immunohistochemical features. Relationships between CT texture features and immunohistochemical markers were assessed by the Spearman correlation test. The diagnostic performance of CT texture parameters in predicting expression levels of immunohistochemical markers was evaluated with ROC analysis. Inter-observer agreement in the measurements of CT texture parameters was estimated with the intra-class correlation coefficient (ICC) (0.000–0.400: poor; 0.401–0.600: moderate; 0.601–0.800: well; 0.801–1.000: excellent). ROC analysis was performed with MedCalc version 15.2.2 statistical software (MedCalc Software bvba, Ostend, Belgium; http://www.medcalc.org; 2015), and other statistical analyses were performed with SPSS (version 22.0 for Microsoft Windows x64, SPSS, Chicago, US). A two-tailed P value less than 0.05 was considered statistically significant.

### Data Availability

The datasets generated and/or analysed during the current study are available from the corresponding author on reasonable request.

## Electronic supplementary material

Supplementary Methods
