# Supplementary material for: CT textural analysis of gastric cancer: correlations with immunohistochemical biomarkers
Source: Sci Rep. 2018 Aug 7;8:11844. doi: 10.1038/s41598-018-30352-6 (PMC6081398; doi:10.1038/s41598-018-30352-6)
Supplement: Supplementary file 1 — Supplementary Methods [file 41598_2018_30352_MOESM1_ESM.docx]

**CT textural analysis of gastric cancer: correlations with immunohistochemical biomarkers**

Shunli Liu^1^, Hua Shi^1^, Changfeng Ji^1^, Wenxian Guan^2^, Ling Chen^3^, Yingshi Sun^4^, Lei Tang^4^, Yue Guan^5^, Weifeng Li^5^, Yun Ge^5^, Jian He^1^, Song Liu^1^, Zhengyang Zhou^1^

1 Department of Radiology, Nanjing Drum Tower Hospital, The Affiliated Hospital of Nanjing University Medical School, Nanjing, China, 210008

2 Department of Gastrointestinal Surgery, Nanjing Drum Tower Hospital, The Affiliated Hospital of Nanjing University Medical School, Nanjing, China, 210008

3 Department of Pathology, Nanjing Drum Tower Hospital, The Affiliated Hospital of Nanjing University Medical School, Nanjing, China, 210008

4 Department of Radiology, Peking University Cancer Hospital & Institute, Beijing, China, 100142

5 School of Electronic Science and Engineering, Nanjing University, Nanjing, China, 210046

First-order entropy was calculated with the following formula:

$$\text{entropy}=-\sum_{i=1}^{G} p_{i}\log_{2}\left(p_{i}\right)$$

G is the number of pixel intensities within the ROI. represents the probability of pixel intensity across the ROI and is computed by dividing the number of the pixel intensity by the total pixel number within the ROI.

Second-order features was calculated with the following formula:

$$\text{entropy(H)}=-\sum_{i=1}^{G} \sum_{j=1}^{G} p\left(i,j\right)\log_{2}\left[p\left(i,j\right)\right]$$

$$\text{correlation}=\sum_{i=1}^{G} \sum_{j=1}^{G} \frac{\left(i-\mu\right)\left(j-\mu\right)p\left(i,j\right)}{\sigma^{2}}$$

$$\text{autocorrelation}=\sum_{i=1}^{G} \sum_{j=1}^{G} ijp(i,j)$$

$$\text{contrast}=\sum_{i=1}^{G} \sum_{j=1}^{G} \left|i-j\right|^{2}p(i,j)$$

G is the number of gray levels within the ROI. $p(i,j)$ represents the probability of a pair of pixels with grey levels $i$ and $j$ occurs in the original image. And those two pixels are spatially dependent in the original image. $\sigma$ is the standard deviation of GLCM element, and $\mu$ is the mean of $p(i,j)$.
